# Competition for waterborne food resources among tropical shallow‐water sponges

**DOI:** 10.1002/ecy.70178

**Published:** 2025-08-10

**Authors:** Mark J. Butler, Steven E. McMurray, Joseph R. Pawlik

**Affiliations:** ^1^ Newfound Harbor Marine Institute Big Pine Key Florida USA; ^2^ Old Woman Creek National Estuarine Research Reserve Huron Ohio USA; ^3^ Department of Biology and Marine Biology, Center for Marine Science University of North Carolina Wilmington North Carolina USA

**Keywords:** Caribbean, competition, Florida, sponge, tropical

## Abstract

A recurrent theme in marine ecology is that the community dynamics of sessile, suspension‐feeding animals is primarily limited by the availability of space, but in some habitats, filtration by these organisms may locally deplete water column resources, setting the stage for exploitative competition for food. We examined filtration by sponge assemblages in the shallow waters (~2 m depth) of Florida Bay (Florida, USA), where water residence times are often high and filtration by dense communities of sponges was hypothesized to deplete the water column of food, primarily picoplankton and dissolved organic matter (DOM). We transplanted three sponge species into replicate locations that differed by an order of magnitude in natural sponge community biomass. Sponge transplants were clones, enabling us to control for sponge genotype effects across all sites. The growth of sponge clones was recorded seasonally for 18–30 months. Growth of transplants placed in areas devoid of sponges was 10 times greater than growth in areas with dense sponge communities and three times greater than growth in areas with average sponge biomass. Sponge mortality was similar regardless of background sponge density. Measurements of picoplankton, DOM, and PO_4_ concentration confirmed an inverse relationship with sponge community biomass, whereas nitrogen concentrations in seawater were highest where sponge species replete with nitrogen‐fixing symbiotic microbial communities were most abundant. This is striking evidence that filtration of waterborne resources by sponges in shallow, coastal environments can deplete those resources sufficiently to cause exploitative competition that limits sponge growth.

## INTRODUCTION

Exploitative competition occurs among species or functional groups with overlapping niches and similar limiting resource requirements (Branch, 1975; Gotelli, 2008). When critical resources are restricted, the consequences of such shortages for inferior competitors can be dramatic, often resulting in decreased growth and recruitment, along with increased mortality (Amundsen et al., 2007; Gross & Cardinale, 2007). Evidence of the negative relationship between population density and growth due to exploitative competition for food or nutrients is founded in Malthusian logic, with widespread examples from bacteria to vertebrates (Candler et al., 2023; Connell, 1983; Damas‐Moreira et al., 2020; Schoener, 1983; Sommer & Worm, 2002).

One of the prevailing paradigms in marine ecology is that populations of sessile suspension‐feeding organisms (Buss & Jackson, 1979; Ferguson et al., 2013; Witman & Dayton, 2001) are primarily limited by competition for limited space on the seafloor (Connell, 1983; Dubois et al., 2007; Lesser et al., 1992), whereas competition for other resources (e.g., food) is considered to be minimal or reduced in importance by predation or physical disturbance (Connell, 1983; Dayton, 1971; Sousa, 2001). This perspective persists despite evidence that removal of growth‐limiting nutrients, food, and even oxygen by suspension‐feeding organisms, including sponges, can result in the depletion of those resources in the water column (Ferguson et al., 2013; Pauly et al., 2022; Peterson & Black, 1987). It thus stands to reason that water column resources could indeed be limiting, especially in oligotrophic ecosystems where their concentrations can decline steeply within dense aggregations of suspension‐feeding organisms (Menge & Sutherland, 1987; Newell, 2004). Evidence for this is strong in bivalves (Cottingham et al., 2023). Laboratory assays indicate that filtering by marine sponges should have powerful effects on water column properties and ecosystem function (Bell, 2008; Gili & Coma, 1998; Perea‐Blazquez et al., 2013; Peterson et al., 2006; Valentine & Butler IV, 2019; van Hoytema et al., 2023), but evidence for this in the field under “real‐world” conditions is lacking.

Sponges, arguably the oldest metazoans (Turner, 2021; Wang et al., 2024), consume suspended particulate organic matter (POM), especially picoplankton in sizes ranging from 0.5 to 50 μm with filtration efficiencies (i.e., particle removal) that typically exceed 75% of the available resources (Hadas et al., 2009; McMurray et al., 2016; Reiswig, 1971; Ribes et al., 1999, 2023; Valentine & Butler IV, 2019). Their removal and conversion of dissolved organic matter (DOM) from seawater is equally as impressive and important (McMurray et al., 2017; Olinger et al., 2021) as that of POM, with the relative consumption of POM and DOM varying among sponge taxa. “High microbial abundance” (HMA) sponge species harbor large concentrations of symbiotic bacteria and consume more DOM than “low microbial abundance” (LMA) species (McMurray et al., 2018; Poppell et al., 2013; Weisz et al., 2007). Where sponges are abundant, their ability to remove particulate and dissolved food from seawater makes them an important benthic‐pelagic link in tropical, temperate, and polar regions (Easson et al., 2015; Hoer et al., 2017; McMurray et al., 2014; Pawlik & McMurray, 2020; Webster & Taylor, 2012). Based on studies describing the effects of sponge filtration on water column constituents, nutrient geochemistry, and habitat provisioning (Archer et al., 2017; Butler IV et al., 1995, 2017; Lynch & Phlips, 2000; Peterson et al., 2006; Valentine & Butler IV, 2019), it is likely that sponges in tropical nearshore habitats are key determinants of ecosystem composition and nutrient cycling.

As predicted by fluid dynamics, the greater the oscula area (i.e., cross‐sectional area of the exhalent siphon) the greater the filtration rate of a sponge (Morganti et al., 2021)—and their filtration can be prodigious. For example, a 1‐kg loggerhead sponge (*Speciospongia vesparium*) can filter 24,000 L of water a day (Vogel, 1994) or 50,000 times their tissue volume (Reiswig, 1971), and while doing so, they remove 6% of the DOM in seawater (Letourneau et al., 2020). The giant barrel sponge (*Xestospongia muta*), a common large sponge on Caribbean reefs, overturns 1.7–12.9 m^3^ of water a day (McMurray et al., 2014). It was estimated that the entire sponge assemblage in Discovery Bay, Jamaica, filtered 15.5–40 m^3^ of the water column per day (Reiswig, 1974). By extrapolating filtration and particle retention rates of sponges in Florida Bay, Peterson et al. (2006) proposed that the loss of the sponge *S. vesparium* in a massive die‐off likely resulted in subsequent cyanobacterial blooms due to reduced filtration by one sponge species.

Despite the magnitude and efficiency of filter feeding by sponges, there is limited evidence that the population dynamics of sponges are driven by food availability. For example, in tropical fore‐reef habitats (>10 m depth), the effect of food limitation on sponges appears minimal presumably because of the turnover of large volumes of water passing over reefs, which provides sponges with a ready supply of POM and DOM (Leichter et al., 2003; Pawlik et al., 2015). Moreover, interspecific competition for attachment space (Wulff, 2006) combined with the presence of spongivorous predators is thought to limit sponge abundance and drive their production of chemical defenses more so than food availability, even where sponges are abundant as they are on many Caribbean reefs (Archer et al., 2020; Leon & Bjorndal, 2002; Loh & Pawlik, 2014; Pawlik, 2011; Pawlik et al., 2015). An exception to the foregoing exists on highly oligotrophic coral reefs in the Red Sea, where there is evidence of food limitation for sponges along a gradient of food availability from nearshore to offshore reefs (Wooster et al., 2019).

By contrast, in tropical nearshore ecosystems where the water is shallow (<3 m) and water residence times are typically longer than on coral reefs (Lee et al., 2016; Leichter et al., 2003; Nuttle et al., 2003), sponges often dominate the sessile animal biomass, with large sponges (>10 cm diameter) sometimes exceeding 80,000 sponges/ha (Butler et al., 2021; Stevely et al., 2010, 2011; Torres et al., 2006). Under such conditions, it is conceivable that filtration by dense assemblages of sponges could deplete the water column of certain fractions of POM and DOM, creating an environment where exploitative competition for food among sponges may ensue (Bacher et al., 2003; Cranford et al., 2008; Peterson et al., 2006).

Here, we present the results of a field study conducted on sponges living in shallow, hard‐bottom habitats on the Florida Bay side of the Florida Keys (Florida, USA). The origins of this study lie in field observations of changes in sponge sizes after widespread sponge die‐offs in Florida Bay (Butler et al., 2021; Butler IV et al., 1995). The few sponges that remained after these die‐offs grew prodigiously to sizes we had never seen before; thus, we surmised that planktonic resources and sponge abundance might interact to drive sponge growth. In this study, we documented the effects of naturally occurring sponge assemblages on nutrient concentrations and plankton communities among sites that varied naturally in sponge biomass and compared the growth of clonal transplants of three sponge species over 18–30 months in those same sites. We hypothesized that sponge growth and water‐column resources would be reduced in sites of high sponge biomass because of sponge filtration and exploitative competition.

## METHODS

### Study sites and experimental setup

**FIGURE 1:**
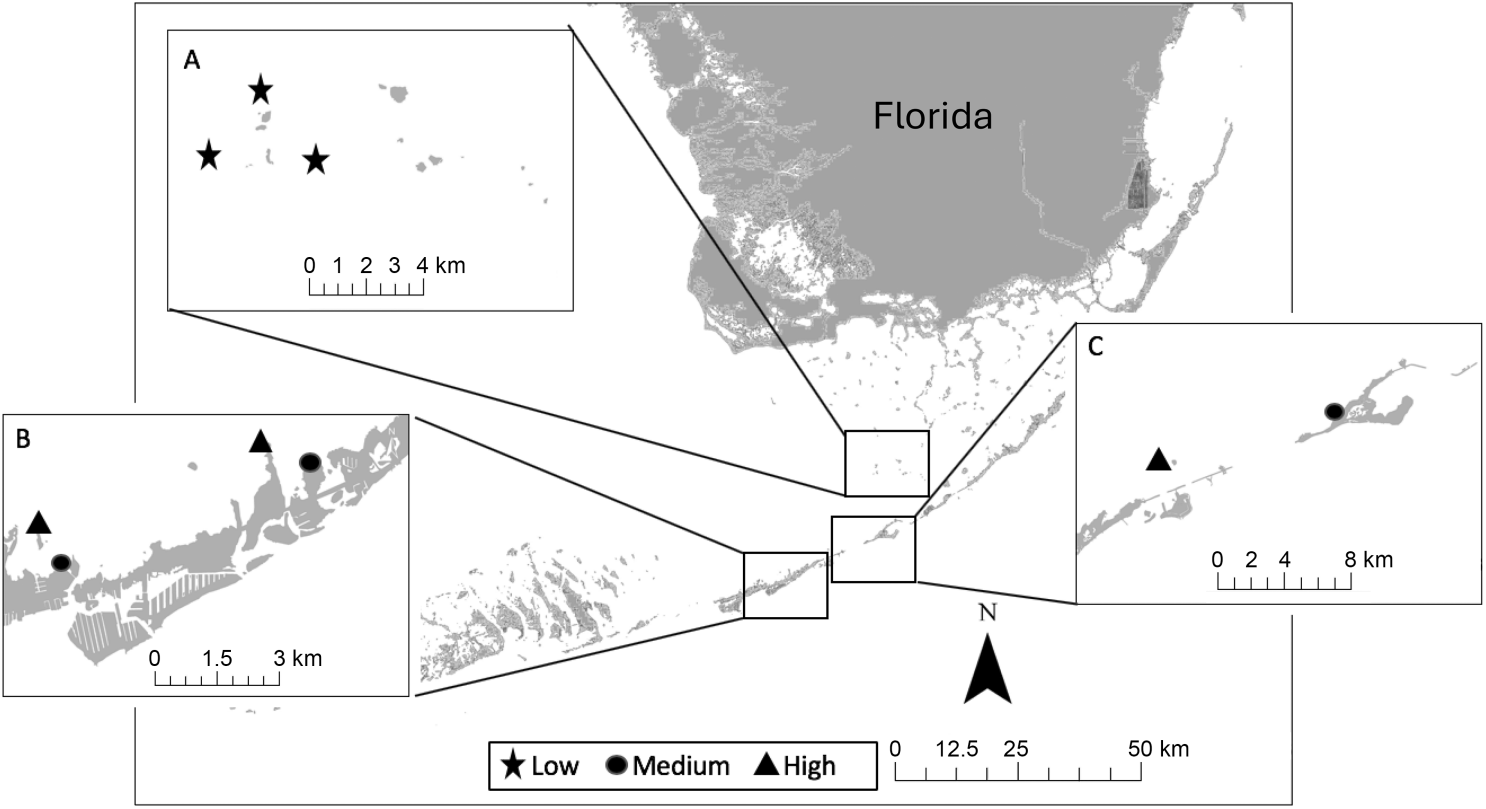
Map of locations (A–C) in the middle Florida Keys, Florida (USA), where sponge clones were out‐planted, with three replicate sites in locations having natural sponge biomass that was either low (star), medium (circle), or high (triangle).

Our study sites were located in the shallow, coastal waters of the Florida Keys (Figure 1), an archipelago south of Florida (USA) where seagrass and hard‐bottom habitats prevail behind a 250‐km long barrier coral reef. Hard‐bottom covers ~30% of the shallow seafloor in the region (Herrnkind et al., 1997; Zieman et al., 1989) and is characterized by low relief, limestone bedrock overlain by a thin veneer of sediment (Chiappone & Sullivan, 1996; Schomer & Drew, 1982). Approximately 60 species of sponges occur in this back‐reef region where their densities can exceed 80,000 sponges/ha with some individuals exceeding 1 m in diameter (Butler et al., 2021; Stevely et al., 2010, 2011; Torres et al., 2006). Depth at our study sites was ~2 m, which is typical for the shallow seas surrounding the Florida Keys, and all sites were separated by a minimum of 0.5 km (Figure 1). Water turnover and vertical mixing in this region of the Florida Keys are largely wind driven (Lee et al., 2016; Nuttle et al., 2003), except near channels where tidal mixing predominates. Our study sites were at least 3 km from channels and experienced similar current velocities (3–12 cm/s) as measured at each site using a WaterMark USGS current meter (Model 6205) during spring tides in March 2016.

**FIGURE 2:**
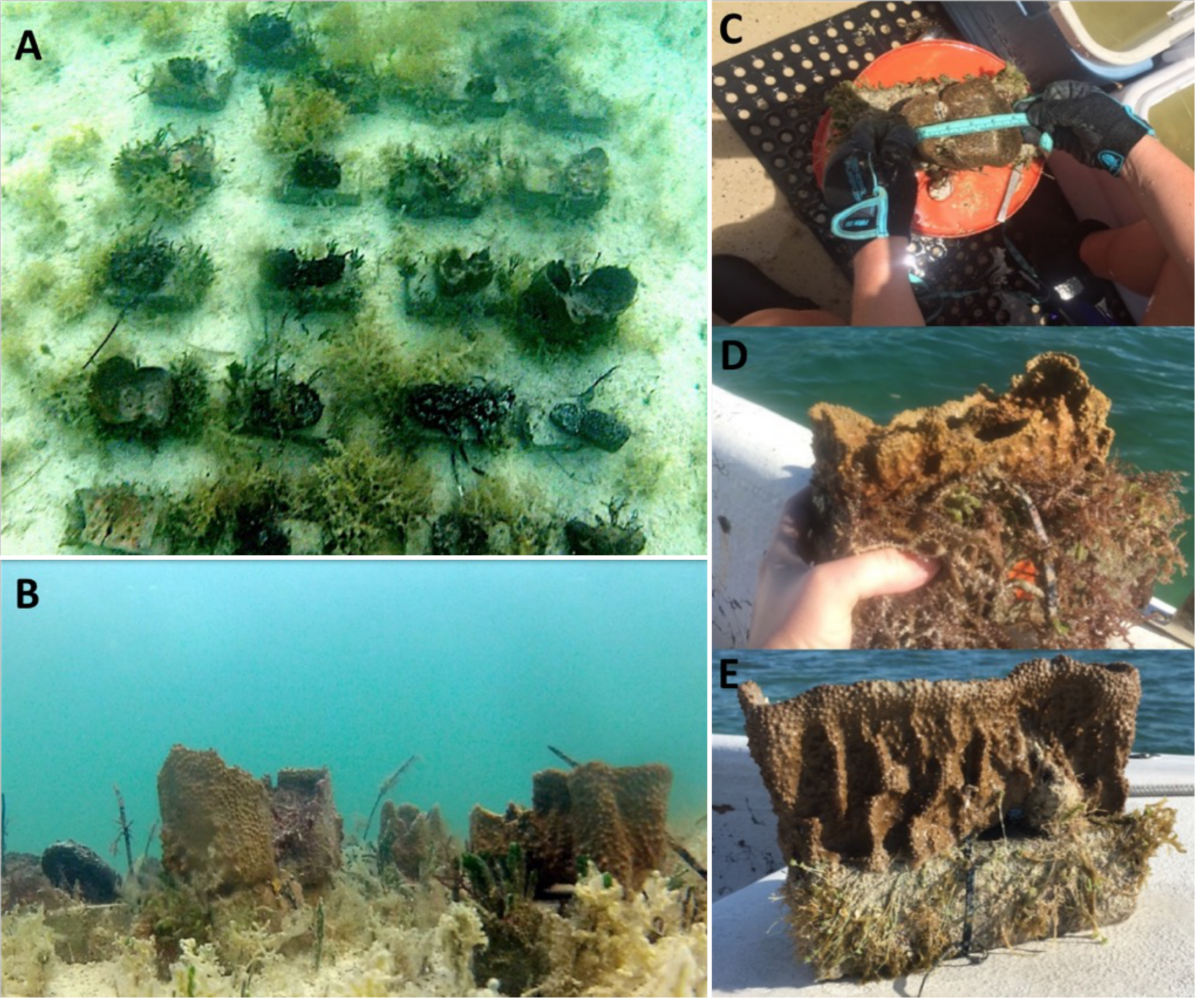
Top (A) and side (B) view photos of transplanted sponges at one of the experimental sites. (C) Measuring a sponge (*Ircinia campana*) clone when first transplanted, and photos of the growth of the same individual after 6 months (D) and 18 months (E). Photo credit: Mark Butler.

We began our study with two species of HMA sponges common to the region, the vase sponge and yellow sponge (*Ircinia campana* and *Spongia barbara*, respectively), which were transplanted into sites having either medium or high natural densities of sponges, and where their growth was monitored for 30 months. Then, 18 months into the study, we added a third sponge species, the grass sponge (*Spongia graminea*). To control for genotype, large sponges of each species were collected from the seafloor and cut into 12–15 smaller pieces (~3500 cm^3^ each) that we attached to brick baseplates with plastic cable ties and numbered tags. These “clones” were left on the sea floor at the collection site for 2 months to heal and affix to the brick baseplates prior to relocation (Figure 2). After the healing period, clones were out‐planted equally into six transplant sites that differed in natural sponge abundance (i.e., medium and high sponge abundance) to test for site‐specific differences in the growth of sponge out‐plants.

Prior to out‐planting sponges, divers surveyed each site to document the diversity, density, and biomass of sponges that naturally occurred at each site. Following established methods for hard‐bottom surveys (Butler IV et al., 1995, 2018; Herrnkind et al., 1997), divers identified and counted each sponge and estimated the volume (biomass) of sponges >20 cm in diameter within four non‐overlapping 25 m × 2 m belt transects positioned haphazardly at each site. Volume estimates were based on sponge shapes and volume formulae appropriate for those shapes (e.g., sphere, cylinder, tube, hollow cone). Prior to out‐planting, the same measurements and formulae were used to estimate the volume of our clone out‐plants. Mean values of volume, density, and diversity were calculated from the four transects per site, and the differences in mean volume were used to designate the sites as high, medium, and low biomass sites.

To begin the experiment, individually tagged sponge clones (14 each of *I. campana* and *S. barbara*) were transplanted in March 2016 in a haphazard arrangement within a 30‐m^2^ area on each of the six medium and high natural sponge density sites. Sponge clones were remeasured and monitored for mortality approximately every 6 months for 18 months: August 2016, March 2017, and August 2017. A year into the study, we added another species, the grass sponge (*S. graminea*), to the study and thereafter continued to monitor the growth and mortality of all three sponge species at the six study sites every 6 months for another 12 months (i.e., a total of 30 months of monitoring for *I. campana* and *S. barbara* and 18 months of monitoring of *S. graminea*). We consider the growth measured each March as indicative of “winter” growth (September–March) and the measurements made in August as indicative of “summer” growth (April–August). During each measurement, sponges and the bricks to which they were attached were cleaned of epizoic organisms. Near the end of the study, we took advantage of a sponge die‐off event caused by a cyanobacterial bloom that killed >90% of the sponges over a large area (500 km^2^) located >15 km from the transplantation sites we were using. Once the die‐off had passed, we tested sponge growth under a third treatment condition by transplanting all three sponge species into this low natural sponge density area (Figure 1). We monitored the growth of sponges transplanted into the low natural sponge density area only once, 6 months after transplantation, because a second sponge‐killing cyanobacterial bloom swept over the same area again, killing all of our transplanted sponges and precluding further measurements.

### Water column characteristics

To document the potential relationship between sponge biomass and water column characteristics at each experimental site, we took water samples at each site in August 2017 at slack tide during a week in which prevailing winds were less than 2.5 m/s. A total of 1 L of seawater was collected in high‐density polyethylene bottles 1 m from the seafloor by sampling 200 mL of water from five locations within each 30‐m^2^ experimental area at each site. Storage and preservation of water samples followed methods presented in McMurray et al. (2016). In the laboratory, water was filtered using pre‐combusted, 0.7‐μm GF/F filters, and 100‐mL aliquots were stored in centrifuge tubes at −20°C for nutrient analysis. Filters were wrapped in aluminum foil and frozen, and 20 mL of filtrate was stored in acidified (100 μL of 50% phosphoric acid) glass vials. Total dissolved nitrogen (TDN) was quantified using an Antek 9000N analyzer that was run in tandem with a Shimadzu TOC 5050 to measure DOC. Particulate organic carbon and nitrogen (POC and PON, respectively) were measured using a CE Elantech NC2100 elemental analyzer. Nutrients (nitrate‐nitrite, ammonia, phosphate) were measured with a Bran+Luebbe AutoAnalyzer III.

For analysis of picoplankton concentrations, 5‐mL seawater subsamples were preserved in electron microscopy grade glutaraldehyde (0.1% final concentration), frozen in liquid nitrogen and after 10 min, stored at −80°C. Phytopicoplankton (picoeukaryotes, *Synechococcus*, *Prochlorococcus*) were enumerated using a BD FACSCelesta Flow Cytometer as previously described (McMurray et al., 2018), and aliquots of each sample were stained with Sybr Green‐1 to quantify high nucleic acid (HNA) bacteria, low nucleic acid (LNA) bacteria, and viruses. Live particulate organic matter (LPOM) as μM carbon and nitrogen for each picoplankton cell type was estimated using standard cell conversions as done in previous studies of sponge feeding (McMurray et al., 2018).

### Statistics

Repeated‐measures general linear models were performed for each sponge species to compare growth across treatments (fixed effect) and sampling events (repeated effect) with site as a random effect. For each species, two separate GLMs were run: (1) one comparing growth under two natural sponge biomass treatment conditions (medium and high) and three sponge species for 18 months (*S. graminea*) or 30 months (*I. campana, S. barbara*) and (2) a second analysis comparing the growth of all three sponge species under three natural sponge biomass treatment conditions (low, medium, and high) after 6 months. Tukey's tests were performed post hoc on significant fixed effects, with Bonferroni corrections applied when interpreting results (i.e., instead of using *p* < 0.05 as the type I error critical value, a result was only considered significant if *p* < 0.004). If an individual died during the experiment, its growth rate was excluded from statistical analysis. A two‐factor crossed GLM followed by a Tukey's test was used to assess whether sponge mortality differed among species or sponge density treatment sites. A one‐factor, fixed‐effects MANOVA was used to compare all water column constituents (11 variables) among the three sponge abundance treatments. Analyses were conducted in SPSS V.30 (IBM Corp).

## RESULTS

### Natural sponge biomass at experimental sites

**FIGURE 3:**
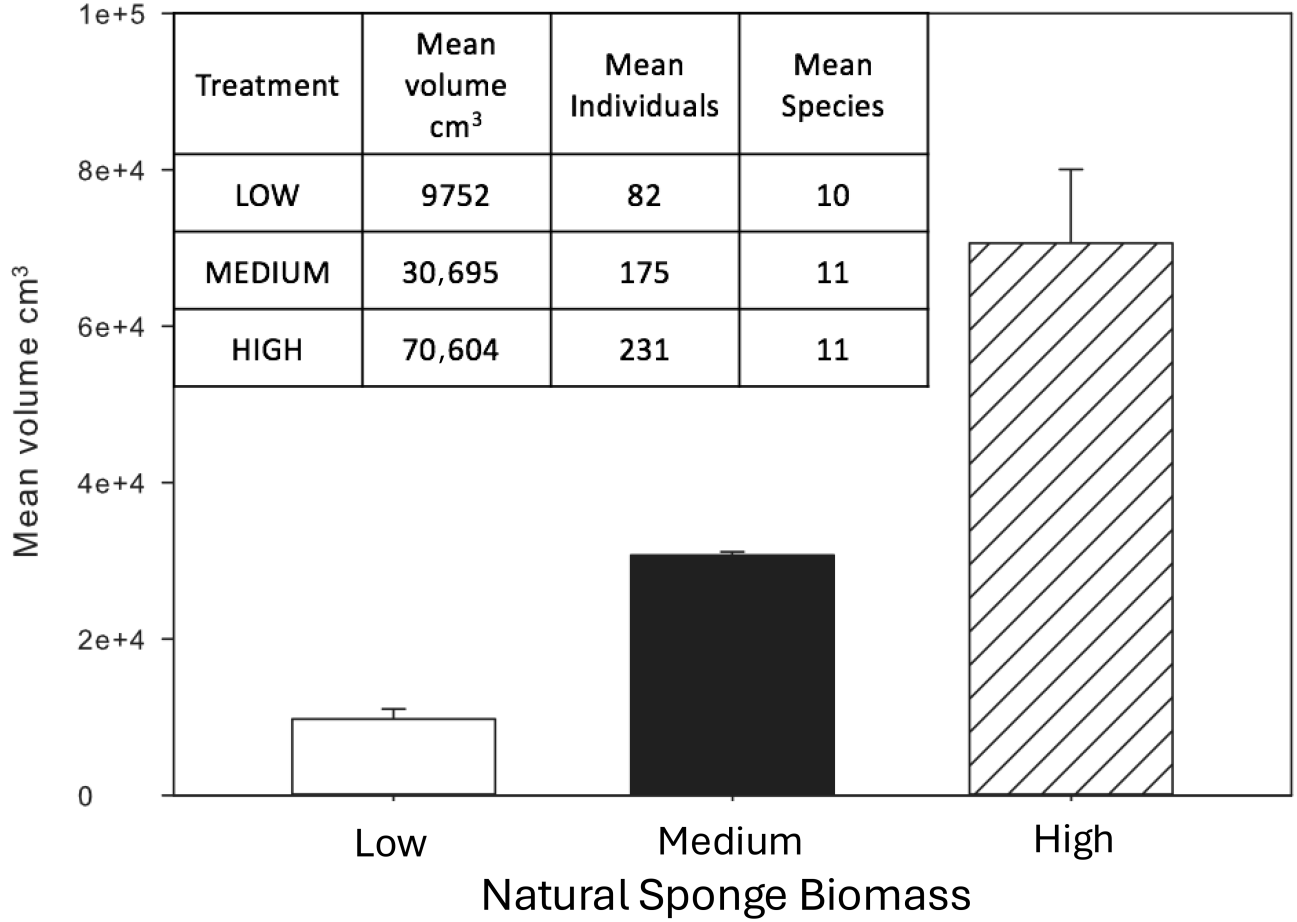
The mean volume of all sponge species at sites designated as low, medium, and high natural sponge biomass treatment sites for subsequent sponge out‐plant experiments (error bars represent ±1SD; *n* = 3 sites). Inset table with mean data on volume (biomass), density, and number of sponge species (diversity) per site for each natural sponge biomass treatment.

There were significant twofold to sevenfold differences in the volume of natural sponges among the high, medium, and low sponge treatment sites (*F* = 63.2; df = 2, 485; *p* < 0.001) in accord with our original classification of the sites (Figure 3). High sponge biomass sites contained an average of 231 (2.31/m^2^) individual sponges >20 cm diameter with a mean sponge biomass of 706 cm^3^/m^2^ from 11 species. At the medium sponge volume site, there was a mean of 174 (1.75/m^2^) sponges with a mean biomass of 307 cm^3^/m^2^ and 11 species. The low sponge volume sites had a mean of only 81 (0.82/m^2^) sponges with a mean biomass of 97 cm^3^/m^2^ from 10 small species.

### Sponge growth by treatment

**FIGURE 4:**
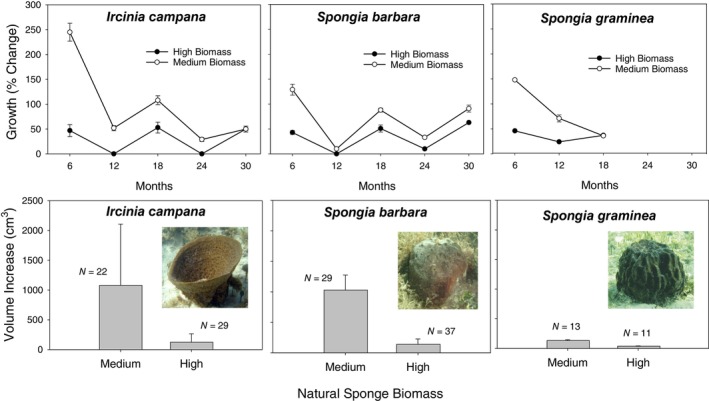
(Top) Mean (±SD) percentage change in growth of three sponge species (*Ircinia campana*, *Spongia barbara*, *Spongia graminea*) over the 30‐month long monitoring period when transplanted to sites with high and medium natural sponge biomass. (Bottom) Total increase in sponge volume of all transplanted sponges of three sponge species when moved to high and medium density sponge sites. Photo credit: Mark Butler.

Regardless of species, growth of sponge clones was inversely proportional to natural sponge biomass, with higher growth in summer months and little or no growth over the winter (Appendix S1: Table S1; Figure 4). Growth of *I. campana* over the 30‐month experiment at high and medium biomass sites averaged 34.7% (*n* = 29) and 135.4% (*n* = 22), respectively. For *S. barbara*, average growth at high and medium biomass sites was 24.5% (*n* = 37) and 79.1% (*n* = 29), respectively. Growth of *S. graminea* at high and medium biomass sites averaged 25.7% (*n* = 11) and 64.5% (*n* = 13), respectively.

The growth of sponges was typically much higher during the spring–summer than during winter, when sponges sometimes shrank in size, which is not unusual. Although increases in sponge volume continued to be significantly greater at the medium sponge biomass sites for both *S. barbara* (*F* = 34.04; df = 1, 26; *p* < 0.0005) and *I. campana* (*F* = 222.541; df = 1, 26; *p* < 0.0005) during the first winter of the experiment and 12 months after deployment, sponge growth slowed dramatically across all species, and in some instances, decreased during winter. Measurements made during the second summer period, 18 months after deployment, showed that the rate of increase in sponge growth was markedly less than that recorded in the first summer period, but it continued to be significantly greater in the medium sponge volume treatment than in the high treatment for *S. barbara* (*F* = 206.73; df = 1, 26; *p* < 0.001) and *I. campana* (*F* = 17.73; df = 1, 26; *p* < 0.001). During the second winter period and after 24 months, changes in sponge volume were again negligible. At the high sponge biomass sites, the volume of sponges decreased rather than increased; however, at the medium sponge biomass sites, there was a small, but significant increase in volume for *S. barbara* (*F* = 132.73; df = 1, 26; *p* < 0.001) and *I. campana* (*F* = 57.87; df = 1, 26; *p* < 0.001). After 30 months, growth of *I. campana* was similar in the two treatments (*F* = 3.10; df = 1, 23; *p* = 0.085), but again significantly greater at medium sponge biomass sites for *S. barbara* (*F* = 132.63; df = 1, 21; *p* < 0.001). In summary, growth of the two species monitored for 30 months was initially high, but tapered off during the course of the study, particularly during winter. However, growth rates were always higher in the medium sponge biomass treatment than in the high sponge biomass treatment, with only one exception (*I. campana* at 30 months).

Twelve months after initial deployment, a third sponge species, *S. graminea*, was added to the experiment, and its patterns of growth were the same as the other species: growth was always significantly greater at the medium sponge biomass sites after 6 months (*F* = 546.96; df = 1, 40; *p* < 0.001), 12 months (*F* = 130.54; df = 1, 40; *p* < 0.001), and 18 months (*F* = 5.67; df = 1, 40; *p* = 0.025).

**FIGURE 5:**
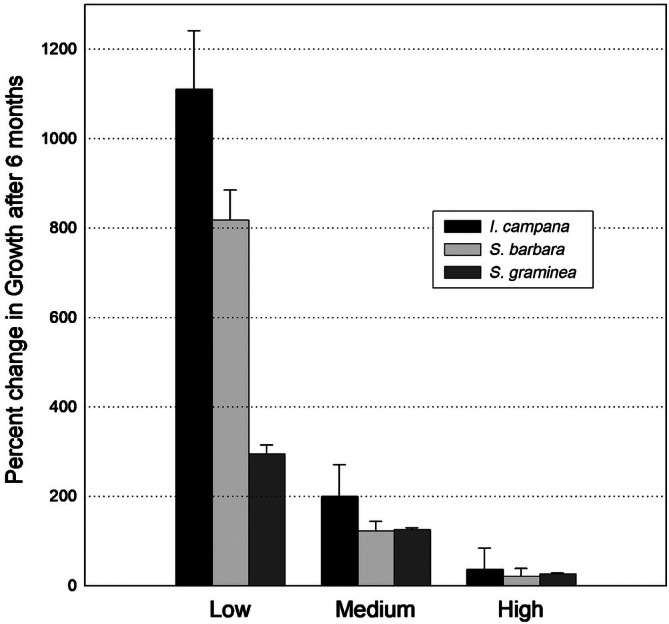
Percentage change in the biomass of three sponge species (*Ircinia campana, Spongia barbara, Spongia graminea*) six months after transplantation into sites with high, medium, and low natural sponge biomass.

A comparison of the growth of all three sponge species at all three natural sponge biomass treatments after 6 months also showed that sponge growth, regardless of species, was inversely related to natural sponge biomass, with the results being most dramatic at the low sponge biomass sites (Appendix S1: Table S2; Figure 5). Growth of two species of sponge transplants (*I. campana, S. barbara*) was approximately an order of magnitude greater at sites where there were few sponges (low biomass treatment) compared to sites with medium to high biomass of sponges (Figure 5), whereas growth of the third species tested (*S. graminea*) doubled at low sponge biomass treatment compared to the medium and high sponge biomass treatments. Mean growth at high, medium, and low sponge biomass sites (respectively) was 36%, 238%, and 1049% for *I. campana*; 30%, 130%, and 353% for *S. barbara*; and 36%, 133%, and 275% for *S. graminea* (Figure 5).

Mortality of sponges did not differ appreciably among sponge biomass treatments (18%–28%; Appendix S1: Table S3; *F* = 3.428; df = 2, 18; *p* = 0.055), but mortality of *I. campana* was higher (34%) than that of *S. barbara* or *S. graminea* (20% and 13%, respectively; *F* = 13.216; df = 2, 18; *p* < 0.001). There was no clear pattern of sponge mortality among species with treatment (i.e., interaction: *F* = 4.240; df = 4, 18; *p* = 0.14; Appendix S1: Figure S1).

### Food availability in the water column

**FIGURE 6:**
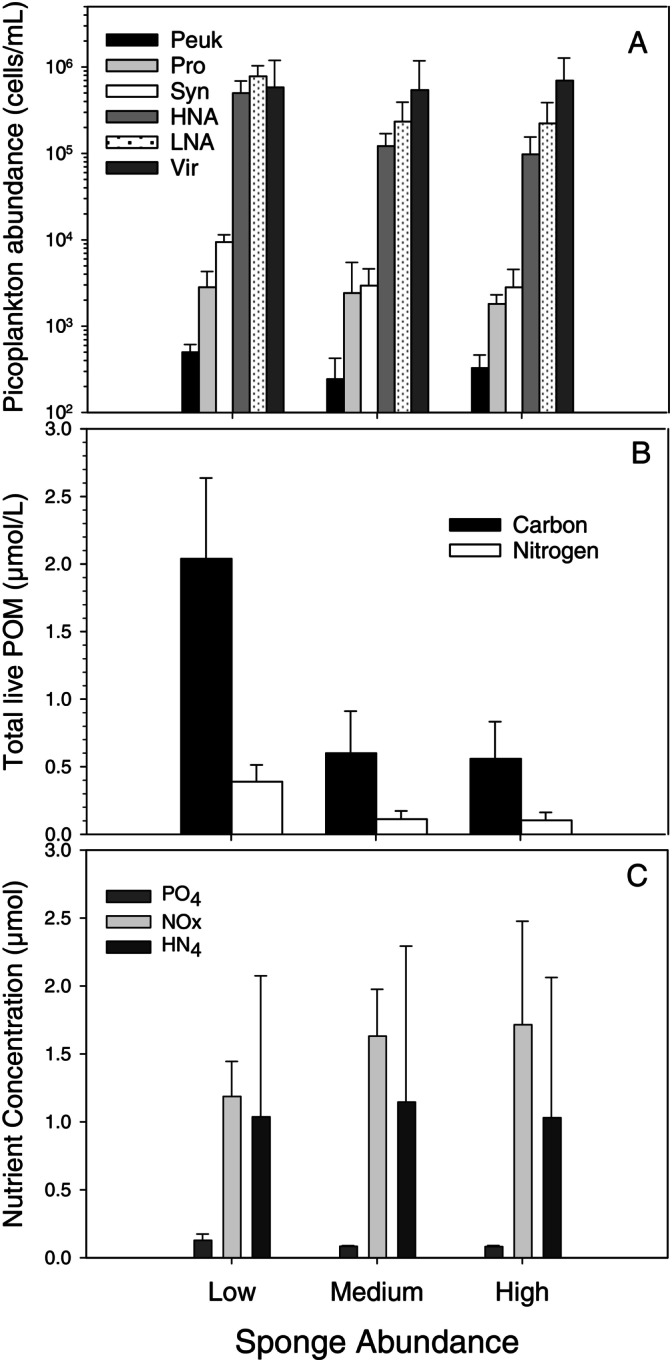
(A) Mean abundance (±SD) of picoplankton in seawater at sites of varying natural sponge biomass (low, medium, high) in the Florida Keys. HNA, high nucleic acid bacteria; LNA, low nucleic acid bacteria; Peuk, picoeukaryotes; Pro, *Prochlorococcus*; Syn, *Synechococcus*; Vir, viruses; note logarithmic scale. (B) Mean concentration (±SD) of total particulate organic matter (POM) in seawater at sites of varying natural sponge biomass. (C) Mean concentration (±SD) of PO_4_^3−^, NO_x_^−^, and NH_4_^+^ at the same sites.

A one‐factor fixed‐effects MANOVA revealed that the three study sites that differed in natural sponge biomass also differed significantly in the concentrations of microbes in the water column (LPOC, i.e., picoeukaryotes, prokaryotes, *Synechococcus*, HNA bacteria, LNA bacteria, viruses) as well as differing significantly in four (POC, PON, PO_4_, NO_x_) of the five seawater chemistry parameters that we measured; ammonia (NH_4_) being the sole exception (Appendix S1: Table S4; Figure 6). Bacterioplankton concentrations (i.e., picoeukaryotes, prokaryotes, *Synechococcus*, HNA bacteria, LNA bacteria), with the exception of viruses, were 2–10 times higher at the low natural sponge biomass sites than at sites where sponges were at medium or high biomass. Similarly, the concentrations of LPOC and PON—aggregate indicators of microplanktonic concentrations—were an order of magnitude higher where sponges were least abundant and lowest at sites where sponges were plentiful. These results are in accord with the predicted effects of ecologically substantial filtering feeding activity by sponges, which, when at high abundance, drive down bacterioplankton concentrations.

Concentrations of PO_4_ and NO_x_ at the low natural sponge biomass sites also differed significantly and meaningfully from those at the medium and high natural sponge biomass sites: PO_4_ was twice as high, and NO_x_ was 30% less at low natural sponge biomass sites compared to medium and high natural sponge biomass sites (Appendix S1: Table S4; Figure 6). Uptake of PO_4_ is consistent with the predicted effects of increasing nutrient uptake by sponges along a gradient of increasing sponge biomass (Archer et al., 2017). The concentration of NO_x_ scaled positively with natural sponge biomass, reflecting the well‐documented nitrogen‐fixing capabilities of symbiotic microbes within the HMA sponges that are the most common at these sites (Fiore et al., 2017; Weisz et al., 2007, 2008). Ammonia concentrations in seawater among the three types of sites did not differ.

## DISCUSSION

The results of this field study indicate that the growth of coastal sponges when at moderate to high natural densities can be limited by waterborne resources. When clones of three species of sponge common in Florida Bay were transplanted into locations that differed in natural sponge community biomass, their subsequent growth differed dramatically and was inversely proportional to the sponge community biomass. Bacterioplankton, NO_x_, and PO_4_ concentrations at the same study sites reflected the strong signature of sponge filtration, in accord with predicted density‐dependent effects. Concentrations of microbes that are the preferred particulate diet of sponges (e.g., picoeukaryotes, cyanobacteria, bacteria) were inversely and non‐linearly correlated with sponge biomass, as was POC and PON. By contrast, NO_x_ concentrations were positively correlated with sponge biomass across locations, a tell‐tale sign of the nitrogen‐fixing capabilities of symbiotic microbes within the HMA sponges that dominate sponge communities in the shallow coastal areas of the Florida Keys. Similarly, concentrations of PO_4_ at low natural sponge biomass sites were twice those at medium and high density sites, mirroring results of other studies of shallow coastal sponge filtration effects (Archer et al., 2017; Valentine & Butler IV, 2019). Sponge mortality did not differ appreciably among natural sponge biomass treatments but varied slightly among species (*S. barbara* at medium natural sponge biomass sites, Appendix S1: Figure S1).

Our findings indicate that there may be an appreciable ecological cost to individual sponges living in dense groups of sponges in shallow‐water environments. What causes high density in these sponge communities? Sponge larvae are generally lecithotrophic and have very short planktonic larval durations, usually on the order of hours to a few days (Lindquist & Hay, 1996; Maldonado & Young, 1996; Maldonado, 2006). Therefore, sponge larval dispersal is limited, especially in shallow coastal areas with little tidal amplitude to transport larvae. As a result, sponge recruitment is localized, and population structure is geographically fragmented (Griffiths et al., 2020; Worheide et al., 2005) compared to other marine taxa with longer planktonic larval durations. Compounding the effects of their limited dispersal is the low availability of rocky, sediment‐free hard‐bottom habitat that settling larvae require for attachment. In short, high densities of sponges and the concomitant detriment to their growth due to exploitative competition for waterborne food resources are likely to be a necessary cost of survival.

Competition is typically greatest between closely related taxa with overlapping resource requirements (e.g., Branch, 1975; Perea‐Blazquez et al., 2013), a condition that would seem to apply to shallow water sponges. In filter‐feeding communities, functional redundancy is assumed to be high (Perea‐Blazquez et al., 2013), but some filter feeders (e.g., ascidians) may partition food resources (Stuart & Klumpp, 1984). Is the same true for sponges? A number of studies have shown that different sponge species (i.e., HMA vs. LMA) differ in their consumption of particle sizes and types of DOM (Lesser et al., 2022; McMurray et al., 2016; Yahel et al., 2003). For example, when Perea‐Blazquez et al. (2013) performed in situ feeding trials in New Zealand with seven species of sponges, they found that each species had different retention efficiencies for different types of picoplankton based on particle size and taxon, an indication of low functional redundancy. However, sponges are rarely found in vast monocultures, as is the case for many filter‐feeding bivalves, so it is likely that differences in their food retention result in increased efficiencies in ecosystem function (e.g., removal or fixation of bacterioplankton and nutrients, benthic‐pelagic cycling) in their more diverse communities. Further, most sponge communities are found at greater depth where the flow of seawater brings unrestricted waterborne food resources that are unlikely to be depleted by re‐processing of seawater by sponges (Pawlik & McMurray, 2020).

A key function of sponge assemblages is their ability to fix or concentrate nutrients such as carbon, nitrogen, and phosphorus (Archer et al., 2017; Valentine & Butler IV, 2019; Webster & Taylor, 2012), much of which can be attributed to the multifaceted relationships of sponges with symbiotic bacteria, archaea, and some eukaryotes (fungi and microalgae). For example, Fiore et al. (2017) applied untargeted and targeted metabolomics techniques to characterize DOM in seawater entering and exiting shallow‐water sponges and found that many more compounds were enhanced in the exhalent samples. Marine ecosystems are often nitrogen limited, but nitrogen is excreted in large quantities by many sponge species (Pita et al., 2018; Valentine & Butler IV, 2019) a product of N‐fixing sponge symbionts (Weisz et al., 2007). The quantities of these compounds released by sponges in nutrient‐limited environments can be ecologically relevant and may stimulate the growth of nearby primary producers (Pawlik et al., 2016; Pita et al., 2018). But the biogeochemical cycling and conversion of waterborne compounds by sponges are far from uniform among species, adding another dimension to the important role of sponge diversity in the potential for exploitative competition and the outcome for ecosystem function.

Despite their inherent logistical difficulties, large‐scale field studies such as ours serve to test the “real‐world” applicability of laboratory results and thus broaden our understanding of processes such as competition that can influence community structure (Stachowicz & Byrnes, 2006). For sponges, the mechanisms structuring communities in tropical habitats such as coral reefs and on mangrove roots are largely attributed to space availability and the presence of spongivores (Pawlik et al., 2018; Wulff, 1997). Spongivores (e.g., angelfishes, parrotfishes) are rare in shallow back‐reef hard‐bottom habitats similar to those studied herein, so that this source of sponge mortality is of little consequence. Indeed, few backreef sponges are chemically defended (Pawlik et al., 1995), reflecting the absence of spongivory as a selective force in these habitats. Instead, our results indicate that the growth of sponges in shallow hard‐bottom areas is limited by competition for waterborne food resources. This conclusion is in keeping with anecdotal observations of the rapid growth of surviving sponges following mass sponge die‐offs in Florida Bay caused by harmful algal blooms (Butler IV et al., 1995; Stevely et al., 2011). Although it may take decades for the sponge communities to recover following a die‐off, especially among climax species, other more “weedy” species (e.g., fire sponge; *Tedania ignis*) that recolonize quickly after a large‐scale community die‐off rapidly grow to unusually large sizes in the absence of other sponges—evidence of competitive release.

Unlike the sponge communities of shallow hard‐bottom habitats considered in the present study, sponge communities on Caribbean fore‐reefs (depth >10 m) are not considered food limited (Pawlik et al., 2015), compared to those found on oligotrophic reefs of the Indo‐Pacific (Wilkinson, 1987) and the Red Sea (Wooster et al., 2019). The nutrition and growth of some reef sponges may also be augmented by symbiotic autotrophs (Erwin & Thacker, 2008; Wilkinson, 1987). By contrast, few sponges that occur in shallow lagoonal habitats contain photosymbionts (e.g., *Ircinia* spp.; Gloeckner et al., 2014). Indeed, in the Caribbean, there are distinct community differences in the distribution of sponge species between fore‐reef and back‐reef lagoonal habitats, perhaps reflecting the drastically different selective pressures in these two habitats. It has been pointed out before that attempts to study coral reef ecosystem function are best conducted on fore‐reef habitats where the complexities of abiotic effects are minimized (reviewed in Pawlik et al., 2018).

Our results indicate that the growth of sponges in shallow hard‐bottom habitats is probably restricted by density‐dependent exploitative competition. We say “probably” because our study is a comparative mensurative experiment rather than a true manipulative experiment (sensu Hurlbert, 1984). That is, we measured a property of the system (i.e., growth of sponge clones) under different natural conditions (i.e., natural sponge biomass) but did not actually manipulate the treatment, which was natural sponge biomass. Doing so at these sites and at the large scales at which our study was done would have been nearly impossible because it would have required the addition or removal of tens of thousands of sponges. However, unlike most mensurative studies, we observed effects of the three treatment conditions (low, medium, and high natural sponge biomass) at three different replicate sites per treatment, each at least 3 km apart and unlikely to differ markedly in background environmental conditions other than natural sponge abundance. In addition, our low natural sponge biomass sites were situated in a large region (>500 km^2^) that had previously supported large, healthy sponge communities before experiencing an episodic, sponge‐killing cyanobacterial bloom. So, there is nothing particularly unique about that region with respect to environmental conditions conducive to sponge growth. In fact, prior to the sponge die‐offs we had surveyed the sponge communities at numerous sites including those where we located our three low sponge biomass treatment sites. The biomass of large (>20 cm diameter) sponges at those three sites before the episodic sponge die‐off ranged from 0.61 to 1.31 sponges/m^2^, which is comparable to what we recorded at our low and medium natural sponge biomass sites (0.82–1.75 sponges/m^2^). This indicates that the sites we chose as low biomass sponge sites had previously supported higher sponge biomass than when we conducted our study. One might argue that water quality conditions may have changed since the sponge die‐offs occurred in 1991, 2007, 2013, and 2017. However, inspection of water quality data collected by the Southeast Environmental Research Center at long‐term, fixed sampling stations (i.e., Old Dan Bank, Bamboo Key, Sprigger Bank) near our various sponge abundance treatment sites does not support that hypothesis. Parameters that are important to sponges such as TOC, TON, and Chl *a* have varied considerably over the available time series and do not show discernable long‐term trends (Appendix S1; Figure 2). The findings of our study are also in accord with mesocosm results conducted under manipulative experimental conditions with the same sponge taxa that demonstrated strong density‐dependent effects of sponges on water column properties (Valentine & Butler IV, 2019). In short, we believe that the data presented here offer convincing evidence of exploitative competition for waterborne food resources among backreef lagoonal sponges.

In summary, our findings highlight the major role that sponge communities play in pelagic‐benthic coupling in shallow coastal environments via their filtration of LPOM and DOM. Dense communities of sponges remove waterborne food resources at rates that are sufficiently high as to result in food limitation and competition. Water column filtration by dense, monospecific beds of bivalves can similarly deplete waterborne food resources in temperate systems (Cottingham et al., 2023). Our work extends this phenomenon to multi‐species sponge communities in shallow tropical hard‐bottom habitats and further demonstrates the negative feedback that ensues at high population densities whereby exploitative competition limits sponge growth.

## AUTHOR CONTRIBUTIONS

Mark J. Butler IV conceived the research, obtained funding for the research, conducted the field research along with graduate research assistants, analyzed the data along with graduate research assistants, and wrote the manuscript. Steven E. McMurray assisted with the field research, processed and analyzed the bacterioplankton data, and edited the manuscript. Joseph R. Pawlik helped interpret the data and edited the manuscript.

## CONFLICT OF INTEREST STATEMENT

The authors declare no conflicts of interest.

## Supporting information


Appendix S1:

## Data Availability

Data (Butler, 2025) are available in Dryad at https://doi.org/10.5061/dryad.02v6wwqb3.
