# Supplementary material for: Competition for waterborne food resources among tropical shallow‐water sponges
Source: Ecology. 2025 Aug 10;106(8):e70178. doi: 10.1002/ecy.70178 (PMC12335821; doi:10.1002/ecy.70178)
Supplement: Supplementary file 1 — Appendix S1: [file ECY-106-e70178-s001.pdf]

APPENDIX S1

Journal: Ecology

Competition for waterborne food resources among tropical shallow-water sponges

Mark J. Butler IV, Steven E. McMurray, and Joseph R. Pawlik

Table S1. ANOVA results for biomass treatment (high, medium, and low natural sponge biomass) and six treatment sites for initial experiment for three selected transplanted sponge species.

Biomass	Sample Period	Sum of Squares	df	Mean Square	F	Sig.
I. campana	6 mos	328771.072	1	328771.072	112.642	<0.001
	12 mos	44670.448	1	44670.448	200.729	<0.001
	18 mos	10604.230	1	10604.230	17.731	<0.001
	24 mos	14000.839	1	14000.839	57.874	<0.001
	30 mos	1247.545	1	1247.545	3.102	0.085
S. barbara	6 mos	159076.426	1	159076.426	618.598	<0.001
	12 mos	3100.304	1	3100.304	34.044	<0.001
	18 mos	31401.323	1	31401.323	206.734	<0.001
	24 mos	9017.185	1	9017.185	132.738	<0.001
	30 mos	31704.680	1	31704.680	132.635	<0.001
S. graminea	6 mos	60398.152	1	60398.152	546.968	<0.001
	12 mos	8702.138	1	8702.138	130.547	<0.001
	18 mos	116.201	1	116.201	5.674	0.025
Site						
I. campana	6 mos	434822.976	5	86964.595	155.486	<0.001
	12 mos	48332.073	5	9666.415	63.074	<0.001
	18 mos	26346.203	5	5269.241	19.936	<0.001
	24 mos	17566.370	5	3513.274	19.852	<0.001
	30 mos	15910.228	5	3182.046	41.985	<0.001
S. barbara	6 mos	164612.761	5	32922.552	179.650	<0.001
	12 mos	7029.204	5	1405.841	43.084	<0.001
	18 mos	34559.675	5	6911.935	62.792	<0.001
	24 mos	11899.404	5	2379.881	94.675	<0.001
	30 mos	39858.912	5	7971.782	65.863	<0.001
S. graminea	6 mos	60856.128	5	12171.226	111.042	<0.001
	12 mos	9753.031	5	1950.606	71.071	<0.001
	18 mos	296.478	5	59.296	3.810	0.014

Table S2. (top table) Results of a 3 x 3 crossed fixed-effect GLM testing the independent and combined effects of sponge species and sponge biomass treatment on the growth of sponges 6 months after transplantation. (bottom table) Tukey HSD results. Species abbreviations used: Ic = *I. campana*; Sb = *S. barbara*; Sg = *S. graminea*.

2-factor fixed-effect GLM

Source	Type III Sum of Squares	df	Mean Square	F	Sig.
Sponge Species	1727886.145	2	863943.073	231.399	<0.0001
Density Treatment	10626026.715	2	5313013.357	1423.040	<0.0001
Species * Treatment	2950747.516	4	737686.879	197.582	<.00001
Error	436827.046	117	3733.564		
Total	28609171.745	126			

Tukey HSD

(I) interaction	(J) interaction	Mean Difference (I-J)	Std. Error	Sig.	95% Confidence Interval	
					Lower Bound	Upper Bound
Ic high	Ic low	-989.9536*	23.09473	<0.001	-1062.9486	-916.9585
	Ic med	-116.0521*	23.09473	<0.001	-189.0472	-43.0571
	Sb high	17.9874	23.09473	0.997	-55.0076	90.9824
	Sb low	-749.1443*	23.09473	<0.001	-822.1393	-676.1492
	Sb med	-43.4738	23.09473	0.627	-116.4689	29.5212
	Sg high	1.2585	23.09473	1.000	-71.7365	74.2536
	Sg low	-196.4710*	23.09473	<0.001	-269.4660	-123.4759
	Sg med	-57.3304	23.09473	0.251	-130.3254	15.6647
Ic low	Ic high	989.9536*	23.09473	<0.001	916.9585	1062.9486
	Ic med	873.9014*	23.09473	<0.001	800.9064	946.8965
	Sb high	1007.9409*	23.09473	<0.001	934.9459	1080.9360
	Sb low	240.8093*	23.09473	<0.001	167.8142	313.8043
	Sb med	946.4797*	23.09473	<0.001	873.4847	1019.4748
	Sg high	991.2121*	23.09473	<0.001	918.2170	1064.2071
	Sg low	793.4826*	23.09473	<0.001	720.4875	866.4776
	Sg med	932.6232*	23.09473	<0.001	859.6281	1005.6182
Ic med	Ic high	116.0521*	23.09473	<0.001	43.0571	189.0472
	Ic low	-873.9014*	23.09473	<0.001	-946.8965	-800.9064
	Sb high	134.0395*	23.09473	<0.001	61.0445	207.0346
	Sb low	-633.0921*	23.09473	<0.001	-706.0872	-560.0971
	Sb med	72.5783	23.09473	0.053	-.4167	145.5734
	Sg high	117.3106*	23.09473	<0.001	44.3156	190.3057
	Sg low	-80.4188*	23.09473	0.019	-153.4139	-7.4238
	Sg med	58.7217	23.09473	0.223	-14.2733	131.7168
Sb high	Ic high	-17.9874	23.09473	0.997	-90.9824	55.0076

	Ic low	-1007.9409*	23.09473	<0.001	-1080.9360	-934.9459
	Ic med	-134.0395*	23.09473	<0.001	-207.0346	-61.0445
	Sb low	-767.1317*	23.09473	<0.001	-840.1267	-694.1366
	Sb med	-61.4612	23.09473	0.174	-134.4562	11.5338
	Sg high	-16.7289	23.09473	0.998	-89.7239	56.2662
	Sg low	-214.4584*	23.09473	<0.001	-287.4534	-141.4633
	Sg med	-75.3178*	23.09473	0.038	-148.3128	-2.3227
Sb low	Ic high	749.1443*	23.09473	<0.001	676.1492	822.1393
	Ic low	-240.8093*	23.09473	<0.001	-313.8043	-167.8142
	Ic med	633.0921*	23.09473	<0.001	560.0971	706.0872
	Sb high	767.1317*	23.09473	<0.001	694.1366	840.1267
	Sb med	705.6704*	23.09473	<0.001	632.6754	778.6655
	Sg high	750.4028*	23.09473	<0.001	677.4077	823.3978
	Sg low	552.6733*	23.09473	<0.001	479.6783	625.6683
	Sg med	691.8139*	23.09473	<0.001	618.8188	764.8089
Sb med	Ic high	43.4738	23.09473	0.627	-29.5212	116.4689
	Ic low	-946.4797*	23.09473	<0.001	-1019.4748	-873.4847
	Ic med	-72.5783	23.09473	0.053	-145.5734	.4167
	Sb high	61.4612	23.09473	0.174	-11.5338	134.4562
	Sb low	-705.6704*	23.09473	<0.001	-778.6655	-632.6754
	Sg high	44.7323	23.09473	0.590	-28.2627	117.7274
	Sg low	-152.9971*	23.09473	<0.001	-225.9922	-80.0021
	Sg med	-13.8566	23.09473	1.000	-86.8516	59.1385
Sg high	Ic high	-1.2585	23.09473	1.000	-74.2536	71.7365
	Ic low	-991.2121*	23.09473	<0.001	-1064.2071	-918.2170
	Ic med	-117.3106*	23.09473	<0.001	-190.3057	-44.3156
	Sb high	16.7289	23.09473	0.998	-56.2662	89.7239
	Sb low	-750.4028*	23.09473	<0.001	-823.3978	-677.4077
	Sb med	-44.7323	23.09473	0.590	-117.7274	28.2627
	Sg low	-197.7295*	23.09473	<0.001	-270.7245	-124.7344
	Sg med	-58.5889	23.09473	0.226	-131.5839	14.4061
Sg low	Ic high	196.4710*	23.09473	<0.001	123.4759	269.4660
	Ic low	-793.4826*	23.09473	<0.001	-866.4776	-720.4875
	Ic med	80.4188*	23.09473	0.019	7.4238	153.4139
	Sb high	214.4584*	23.09473	<0.001	141.4633	287.4534
	Sb low	-552.6733*	23.09473	<0.001	-625.6683	-479.6783
	Sb med	152.9971*	23.09473	<0.001	80.0021	225.9922
	Sg high	197.7295*	23.09473	<0.001	124.7344	270.7245
	Sg med	139.1406*	23.09473	<0.001	66.1455	212.1356
Sg med	Ic high	57.3304	23.09473	0.251	-15.6647	130.3254
	Ic low	-932.6232*	23.09473	<0.001	-1005.6182	-859.6281
	Ic med	-58.7217	23.09473	0.223	-131.7168	14.2733
	Sb high	75.3178*	23.09473	0.038	2.3227	148.3128

	Sb low	-691.8139*	23.09473	<0.001	-764.8089	-618.8188
	Sb med	13.8566	23.09473	1.000	-59.1385	86.8516
	Sg high	58.5889	23.09473	0.226	-14.4061	131.5839
	Sg low	-139.1406*	23.09473	<0.001	-212.1356	-66.1455

Table S3: (top table) Results of a 3 x 3 crossed fixed-effect GLM testing the independent and combined effects of sponge species and sponge biomass treatment on the mortality of sponges 18 months after transplantation. (bottom table) Tukey HSD results. Species abbreviations used: Ic = *I. campana*; Sb = *S. barbara*; Sg = *S. graminea*

Source	Type III Sum of Squares	df	Mean Square	F	Sig.
Species	1993.185	2	996.593	13.216	<0.001
Density Treatment	516.963	2	258.481	3.428	0.055
Species * Treatment	1278.815	4	319.704	4.240	0.014
Error	1357.333	18	75.407		
Total	166773.000	27			

Tukey HSD Results

(I) treatment	(J) treatment	Mean Difference (I-J)	Std. Error	Sig.	95% Confidence Interval Lower Bound	Upper Bound
Ic high	Ic low	-7.6667	7.09025	0.970	-32.5099	17.1766
	Ic med	16.0000	7.09025	0.414	-8.8432	40.8432
	Sb high	-19.3333	7.09025	0.206	-44.1766	5.5099
	Sb low	-13.6667	7.09025	0.605	-38.5099	11.1766
	Sb med	.0000	7.09025	1.000	-24.8432	24.8432
	Sg high	-10.0000	7.09025	0.880	-34.8432	14.8432
	Sg low	-19.0000	7.09025	0.222	-43.8432	5.8432
	Sg med	-24.6667	7.09025	0.052	-49.5099	.1766
Ic low	Ic high	7.6667	7.09025	0.970	-17.1766	32.5099
	Ic med	23.6667	7.09025	0.069	-1.1766	48.5099
	Sb high	-11.6667	7.09025	0.769	-36.5099	13.1766
	Sb low	-6.0000	7.09025	0.993	-30.8432	18.8432
	Sb med	7.6667	7.09025	0.970	-17.1766	32.5099
	Sg high	-2.3333	7.09025	1.000	-27.1766	22.5099
	Sg low	-11.3333	7.09025	0.794	-36.1766	13.5099
	Sg med	-17.0000	7.09025	0.341	-41.8432	7.8432
Ic med	Ic high	-16.0000	7.09025	0.414	-40.8432	8.8432
	Ic low	-23.6667	7.09025	0.069	-48.5099	1.1766
	Sb high	-35.3333*	7.09025	0.002	-60.1766	-10.4901
	Sb low	-29.6667*	7.09025	0.013	-54.5099	-4.8234
	Sb med	-16.0000	7.09025	0.414	-40.8432	8.8432
	Sg high	-26.0000*	7.09025	0.036	-50.8432	-1.1568

	Sg low	-35.0000*	7.09025	0.003	-59.8432	-10.1568
	Sg med	-40.6667*	7.09025	<.001	-65.5099	-15.8234
Sb high	Ic high	19.3333	7.09025	0.206	-5.5099	44.1766
	Ic low	11.6667	7.09025	0.769	-13.1766	36.5099
	Ic med	35.3333*	7.09025	0.002	10.4901	60.1766
	Sb low	5.6667	7.09025	0.995	-19.1766	30.5099
	Sb med	19.3333	7.09025	0.206	-5.5099	44.1766
	Sg high	9.3333	7.09025	0.914	-15.5099	34.1766
	Sg low	.3333	7.09025	1.000	-24.5099	25.1766
	Sg med	-5.3333	7.09025	0.997	-30.1766	19.5099
Sb low	Ic high	13.6667	7.09025	0.605	-11.1766	38.5099
	Ic low	6.0000	7.09025	0.993	-18.8432	30.8432
	Ic med	29.6667*	7.09025	0.013	4.8234	54.5099
	Sb high	-5.6667	7.09025	0.995	-30.5099	19.1766
	Sb med	13.6667	7.09025	0.605	-11.1766	38.5099
	Sg high	3.6667	7.09025	1.000	-21.1766	28.5099
	Sg low	-5.3333	7.09025	0.997	-30.1766	19.5099
	Sg med	-11.0000	7.09025	0.817	-35.8432	13.8432
Sb med	Ic high	.0000	7.09025	1.000	-24.8432	24.8432
	Ic low	-7.6667	7.09025	0.970	-32.5099	17.1766
	Ic med	16.0000	7.09025	0.414	-8.8432	40.8432
	Sb high	-19.3333	7.09025	0.206	-44.1766	5.5099
	Sb low	-13.6667	7.09025	0.605	-38.5099	11.1766
	Sg high	-10.0000	7.09025	0.880	-34.8432	14.8432
	Sg low	-19.0000	7.09025	0.222	-43.8432	5.8432
	Sg med	-24.6667	7.09025	0.052	-49.5099	.1766
Sg high	Ic high	10.0000	7.09025	0.880	-14.8432	34.8432
	Ic low	2.3333	7.09025	1.000	-22.5099	27.1766
	Ic med	26.0000*	7.09025	0.036	1.1568	50.8432
	Sb high	-9.3333	7.09025	0.914	-34.1766	15.5099
	Sb low	-3.6667	7.09025	1.000	-28.5099	21.1766
	Sb med	10.0000	7.09025	0.880	-14.8432	34.8432
	Sg low	-9.0000	7.09025	0.928	-33.8432	15.8432
	Sg med	-14.6667	7.09025	0.521	-39.5099	10.1766
Sg low	Ic high	19.0000	7.09025	0.222	-5.8432	43.8432
	Ic low	11.3333	7.09025	0.794	-13.5099	36.1766
	Ic med	35.0000*	7.09025	0.003	10.1568	59.8432
	Sb high	-.3333	7.09025	1.000	-25.1766	24.5099

	Sb low	5.3333	7.09025	0.997	-19.5099	30.1766
	Sb med	19.0000	7.09025	0.222	-5.8432	43.8432
	Sg high	9.0000	7.09025	0.928	-15.8432	33.8432
	Sg med	-5.6667	7.09025	0.995	-30.5099	19.1766
Sg med	Ic high	24.6667	7.09025	0.052	-.1766	49.5099
	Ic low	17.0000	7.09025	0.341	-7.8432	41.8432
	Ic med	40.6667*	7.09025	<.001	15.8234	65.5099
	Sb high	5.3333	7.09025	0.997	-19.5099	30.1766
	Sb low	11.0000	7.09025	0.817	-13.8432	35.8432
	Sb med	24.6667	7.09025	0.052	-.1766	49.5099
	Sg high	14.6667	7.09025	0.521	-10.1766	39.5099
	Sg low	5.6667	7.09025	0.995	-19.1766	30.5099

Based on observed means. The error term is Mean Square(Error) = 75.407.

Table S4: Statistical results comparing water quality parameters among the three study sites: low, medium, and high natural sponge biomass. (top table) Results of 1-factor fixed-effect MANOVA testing overall differences among study sites for 11 water quality parameters: picoeukaryotes, (Peuk), *Prochlorococcus* (Pro), *Synechococcus*, (Syn), high nucleic acid bacteria (HNA), low nucleic acid bacteria (LNA), viruses (Virus), particulate organic carbon (POC), particulate organic matter (POM), orthophosphate (PO_4), nitrate-nitrite (NO_x), ammonia (NH_4). (middle table) Results of separate1-factor fixed-effect ANOVAs testing differences among study sites for each water quality parameter. (bottom table) Pairwise Tukey HSD multiple comparison test results for each ANOVA.

MANOVA Results					
Statistic	Value	F	Hypothesis df	Error df	Sig.
Wilk's Lambda	0.000033	28.538	12	2	0.034
Roy's Largest Root	2437.445	812.482	12	2	0.001
Pillai's Trace	1.917	7.736	6	2	0.031

ANOVA Results

Source	Dependent Variable	Type III Sum of Squares	df	Mean Square	F	Sig.
Sponge Density	Peuk	87501.556	2	43750.778	46.637	<0.001
	Pro	2173437.556	2	1086718.778	54.131	<0.001
	Syn	84986040.889	2	42493020.444	100.209	<0.001
	HNA	161608988696.889	2	80804494348.444	119.176	<0.001
	LNA	600619278206.889	2	300309639103.444	282.069	<0.001
	Virus	11801197040.667	2	5900598520.333	8.195	0.019
	POC	4.482	2	2.241	59.916	<0.001
	PON	.300	2	.150	173.192	<0.001
	PO4	.021	2	.010	19.809	0.002
	NOx	.613	2	.307	8.325	0.019
	NH4	.137	2	.069	4.245	0.071
Error	Peuk	5628.667	6	938.111		
	Pro	120453.333	6	20075.556		
	Syn	2544272.667	6	424045.444		
	HNA	4068168636.667	6	678028106.111		
	LNA	6387997074.667	6	1064666179.111		
	Virus	4320117215.333	6	720019535.889		
	POC	.224	6	.037		
	PON	.005	6	.001		
	PO4	.003	6	.001		
	NOx	.221	6	.037		
	NH4	.097	6	.016		
Total	Peuk	694789.000	9			
	Pro	20012378.000	9			
	Syn	290868407.000	9			
	HNA	347657607403.000	9			

	LNA	1413421452647.000	9			
	Virus	2137166931417.000	9			
	POC	14.608	9			
	PON	.741	9			
	PO4	.189	9			
	NOx	24.519	9			
	NH4	11.037	9			

Tukey HSD Results

Dependent Variable	Sponge Density (I)	Sponge Density (J)	Mean Difference (I-J)	Sig.	95% Confidence Interval Lower Bound	Upper Bound
Peuk	high	low	-183.6667*	<0.001	-260.3985	-106.9348
		med	44.0000	0.260	-32.7319	120.7319
	low	high	183.6667*	<0.001	106.9348	260.3985
		med	227.6667*	<0.001	150.9348	304.3985
	med	high	-44.0000	0.260	-120.7319	32.7319
		low	-227.6667*	<0.001	-304.3985	-150.9348
Pro	high	low	-1185.0000*	<0.001	-1539.9624	-830.0376
		med	-409.3333*	0.028	-764.2958	-54.3709
	low	high	1185.0000*	<0.001	830.0376	1539.9624
		med	775.6667*	<0.001	420.7042	1130.6291
	med	high	409.3333*	0.028	54.3709	764.2958
		low	-775.6667*	<0.001	-1130.6291	-420.7042
Syn	high	low	-6520.0000*	<0.001	-8151.3787	-4888.6213
		med	-2.6667	1.000	-1634.0454	1628.7121
	low	high	6520.0000*	<0.001	4888.6213	8151.3787
		med	6517.3333*	<0.001	4885.9546	8148.7121
	med	high	2.6667	1.000	-1628.7121	1634.0454
		low	-6517.3333*	<0.001	-8148.7121	-4885.9546
HNA	high	low	-235932.0000*	<0.001	-301165.7650	-170698.2350
		med	79661.3333*	0.022	14427.5683	144895.0984
	low	high	235932.0000*	<0.001	170698.2350	301165.7650
		med	315593.3333*	<0.001	250359.5683	380827.0984
	med	high	-79661.3333*	0.022	-144895.0984	-14427.5683
		low	-315593.3333*	<0.001	-380827.0984	-250359.5683
LNA	high	low	-550848.3333*	<0.001	-632592.2280	-469104.4387
		med	-5731.3333	0.975	-87475.2280	76012.5613
	low	high	550848.3333*	<0.001	469104.4387	632592.2280
		med	545117.0000*	<0.001	463373.1053	626860.8947
	med	high	5731.3333	0.975	-76012.5613	87475.2280
		low	-545117.0000*	<0.001	-626860.8947	-463373.1053
Virus	high	low	45337.6667	0.177	-21885.7739	112561.1073
		med	88691.3333*	0.016	21467.8927	155914.7739

	low	high	-45337.6667	0.177	-112561.1073	21885.7739
		med	43353.6667	0.198	-23869.7739	110577.1073
	med	high	-88691.3333*	0.016	-155914.7739	-21467.8927
		low	-43353.6667	0.198	-110577.1073	23869.7739
POC	high	low	-1.5133*	<0.001	-1.9978	-1.0288
		med	-.0333	0.976	-.5178	.4512
	low	high	1.5133*	<0.001	1.0288	1.9978
		med	1.4800*	<0.001	.9955	1.9645
	med	high	.0333	0.976	-.4512	.5178
		low	-1.4800*	<0.001	-1.9645	-.9955
PON	high	low	-.3600*	<0.001	-.4338	-.2862
		med	.0500	0.174	-.0238	.1238
	low	high	.3600*	<0.001	.2862	.4338
		med	.4100*	<0.001	.3362	.4838
	med	high	-.0500	0.174	-.1238	.0238
		low	-.4100*	<0.001	-.4838	-.3362
PO4	high	low	-.1000*	0.004	-.1573	-.0427
		med	.0033	0.983	-.0539	.0606
	low	high	.1000*	0.004	.0427	.1573
		med	.1033*	0.004	.0461	.1606
	med	high	-.0033	0.983	-.0606	.0539
		low	-.1033*	0.004	-.1606	-.0461
NOx	high	low	.5933*	0.021	.1125	1.0742
		med	.0900	0.838	-.3909	.5709
	low	high	-.5933*	0.021	-1.0742	-.1125
		med	-.5033*	0.042	-.9842	-.0225
	med	high	-.0900	0.838	-.5709	.3909
		low	.5033*	0.042	.0225	.9842
NH4	high	low	-.2000	0.212	-.5186	.1186
		med	-.2967	0.065	-.6153	.0220
	low	high	.2000	0.212	-.1186	.5186
		med	-.0967	0.642	-.4153	.2220
	med	high	.2967	0.065	-.0220	.6153
		low	.0967	0.642	-.2220	.4153

Homogeneous Subsets by Species

Tukey HSD[a,b]

species	N	Subset 1	2
I.campana	9	65.8889	
S.barbara	9		79.6667

S.cheris	9		86.5556
Sig.		1.000	.239

Means for groups in homogeneous subsets are displayed.
Based on observed means.
The Mean Square (Error) = 75.407.
Alpha P = .05.

Homogeneous Subsets by Treatment

Tukey HSD[a,b]

		Subset	
treatment	N	1	2
medium	9	71.5556	
high	9	78.4444	78.4444
low	9		82.1111
Sig.		.239	.650

Means for groups in homogeneous subsets are displayed.
Based on observed means.
The Mean Square (Error) = 75.407.
Alpha p = .05.

Figure S1: Graph showing the interactive effect of sponge species (*S. barbara, I. campana, S. cheris* = *S. graminea*) and natural sponge biomass treatment (low, medium, high) on sponge transplant survival. Plotted are means + 95 CI.

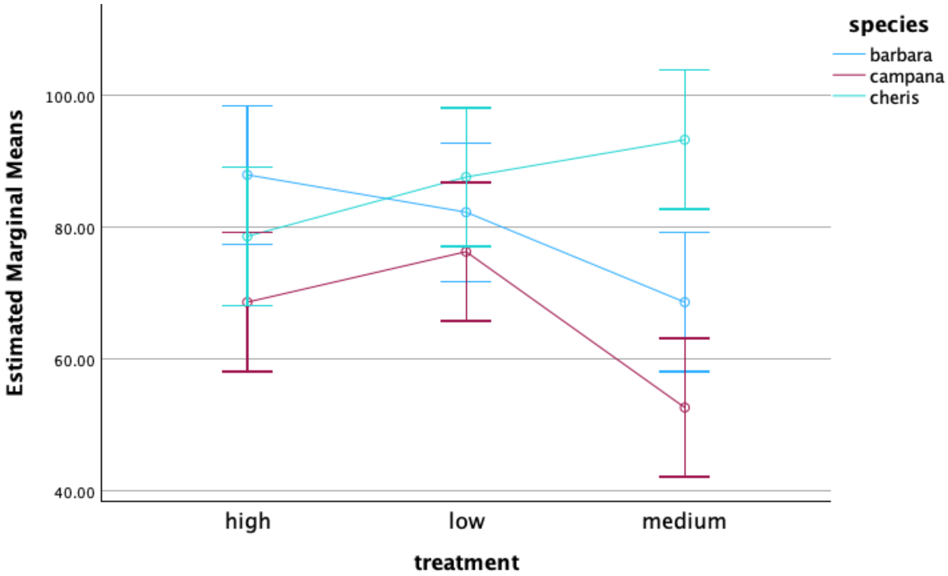

Figure S2: Time-series (1995 – 2003) of water quality data (TOC, Chl a, TON, NO_3, TP) at three locations in the general area of our study sites. (Southeast Environmental Research Center 2025).

Station Number 289
Area SLU
Site Name Bamboo Key
Latitude Degrees 24 46.665
Longitude Degrees -81 01.980

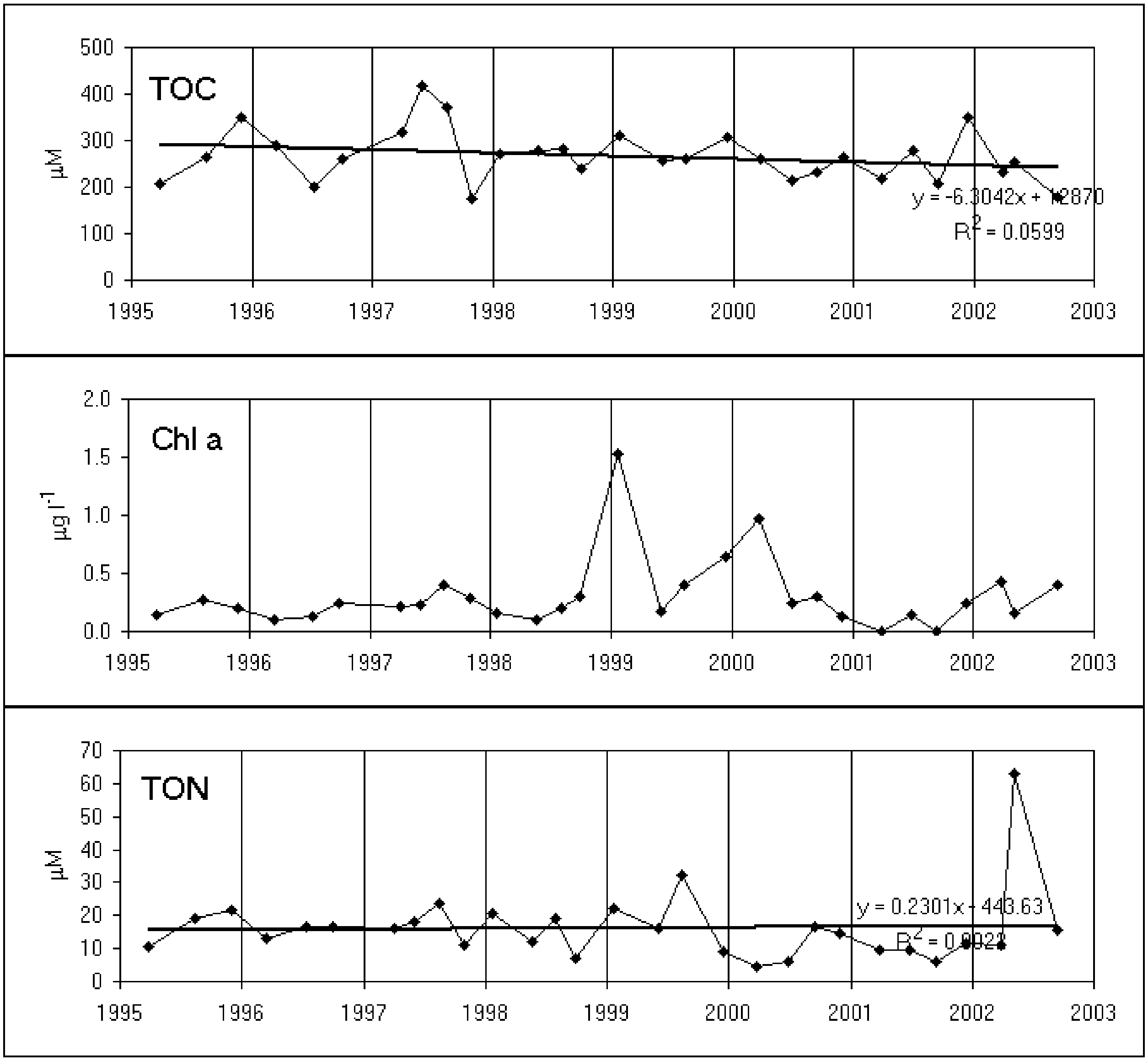

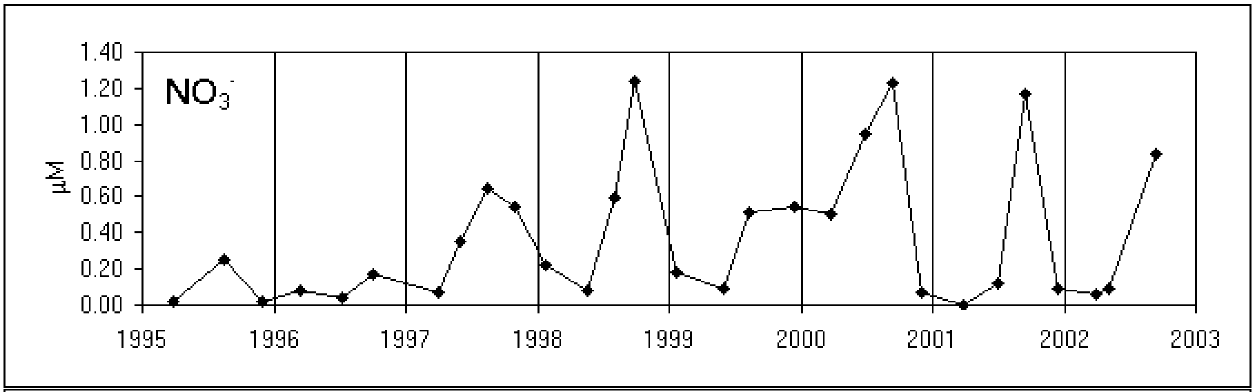

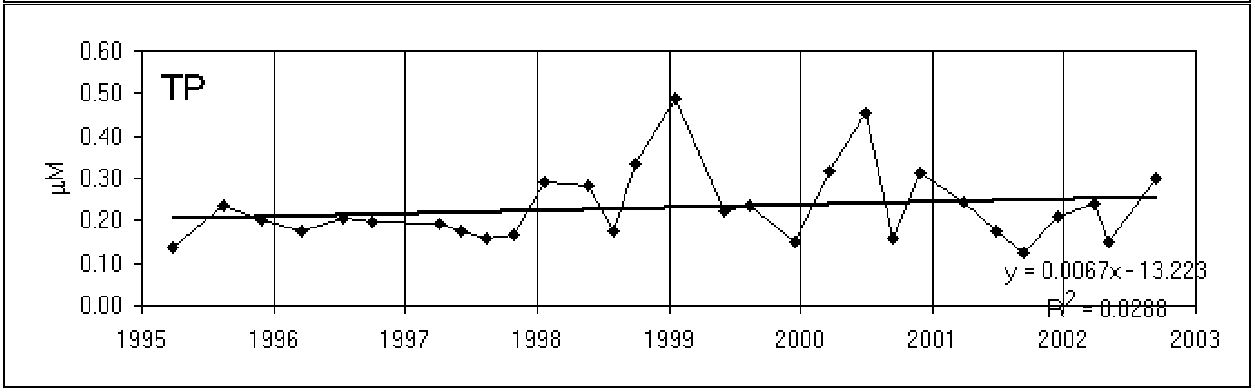

**Station Number**	28
**Area**	FLBAY
**Site Name**	Old Dan Bank
**Latitude Degrees**	24 52.032
**Longitude Degrees**	-80 48.429

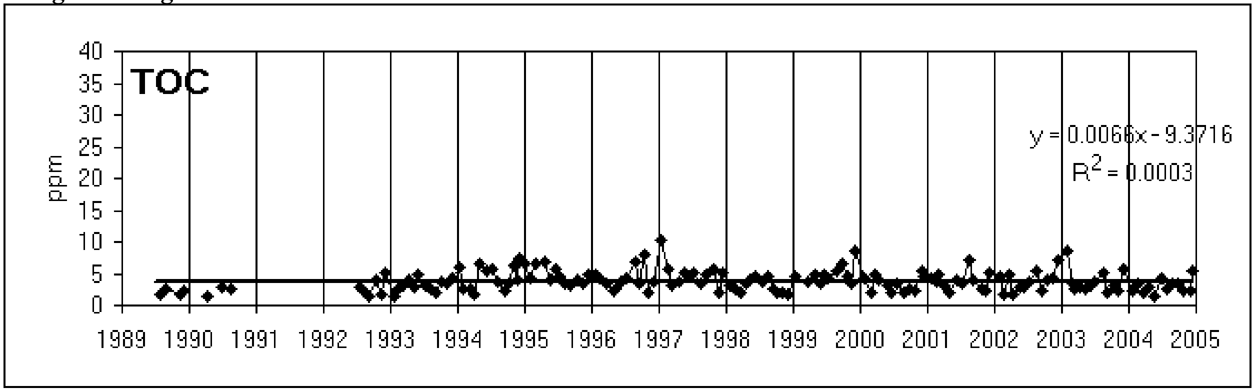

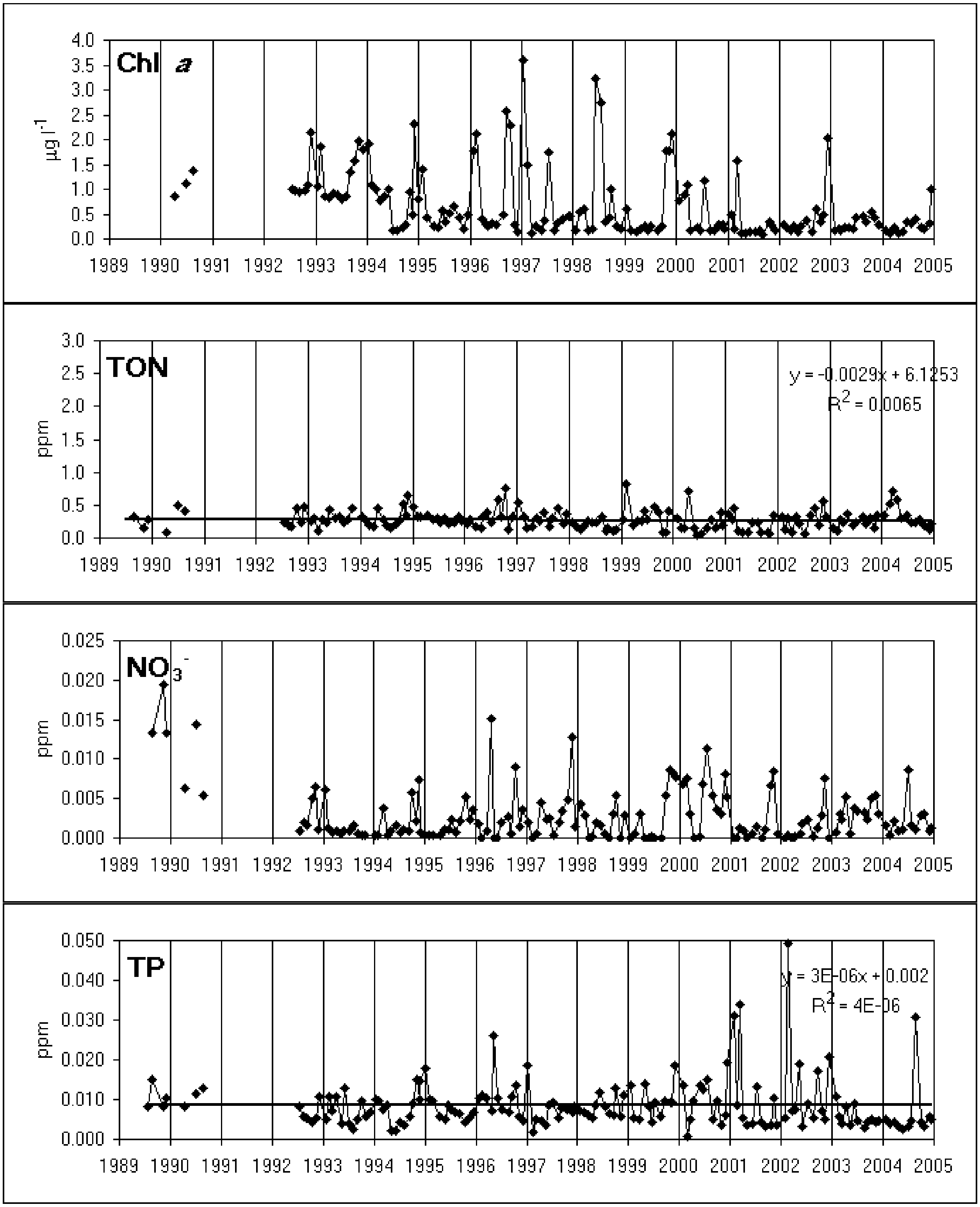

Chl a
µg l-1
4.0
3.5
3.0
2.5
2.0
1.5
1.0
0.5
0.0
1989 1990 1991 1992 1993 1994 1995 1996 1997 1998 1999 2000 2001 2002 2003 2004 2005
TON
ppm
3.0
2.5
2.0
1.5
1.0
0.5
0.0
y = -0.0029x + 6.1253
R2 = 0.0065
1989 1990 1991 1992 1993 1994 1995 1996 1997 1998 1999 2000 2001 2002 2003 2004 2005
NO3-
ppm
0.025
0.020
0.015
0.010
0.005
0.000
1989 1990 1991 1992 1993 1994 1995 1996 1997 1998 1999 2000 2001 2002 2003 2004 2005
TP
ppm
0.050
0.040
0.030
0.020
0.010
0.000
y = 3E-06x + 0.002
R2 = 4E-06
1989 1990 1991 1992 1993 1994 1995 1996 1997 1998 1999 2000 2001 2002 2003 2004 2005

**Station Number**	27
**Area**	FLBAY
**Site Name**	Sprigger Bank
**Latitude Degrees**	24 55.116
**Longitude Degrees**	-80 56.092

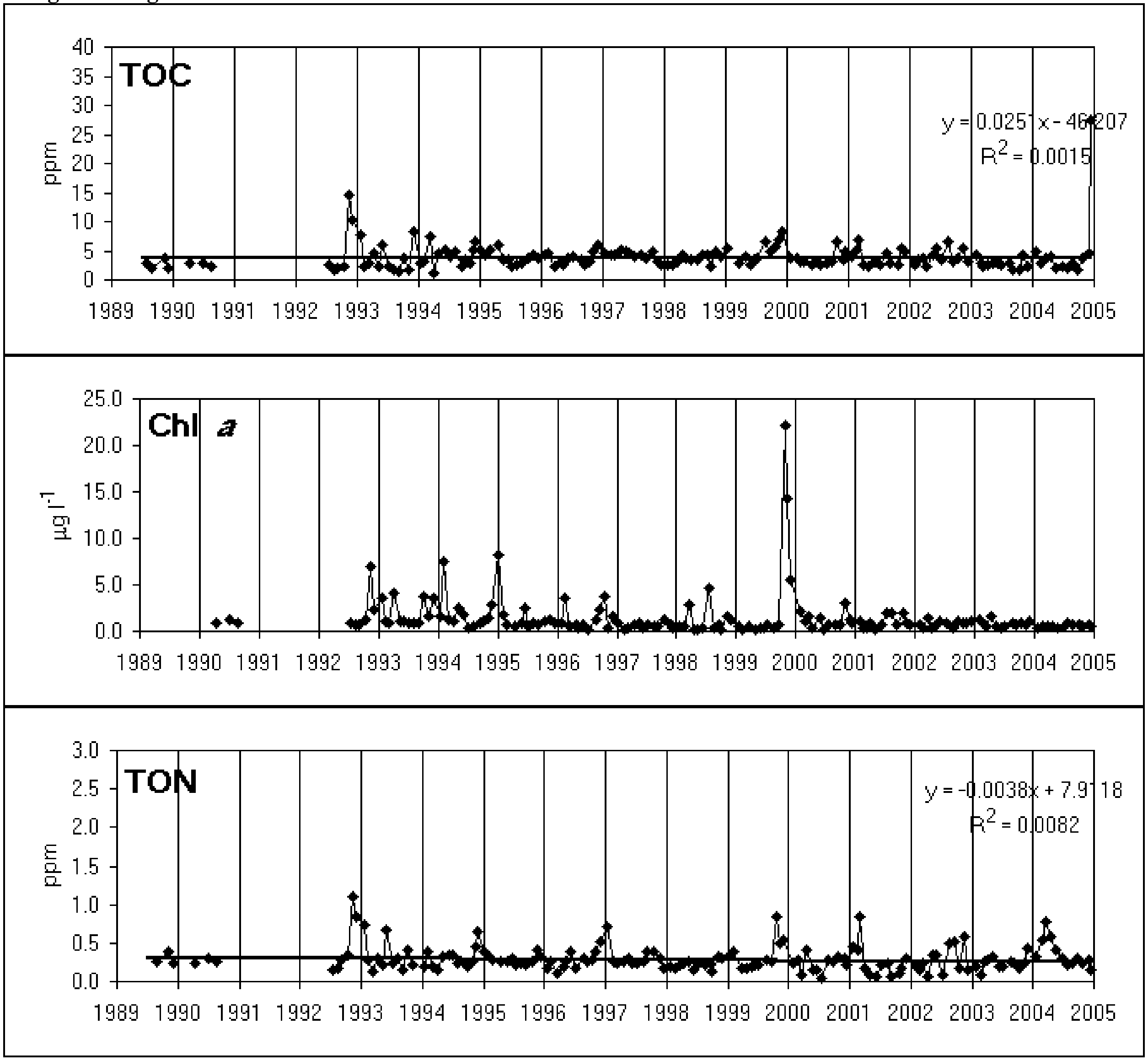

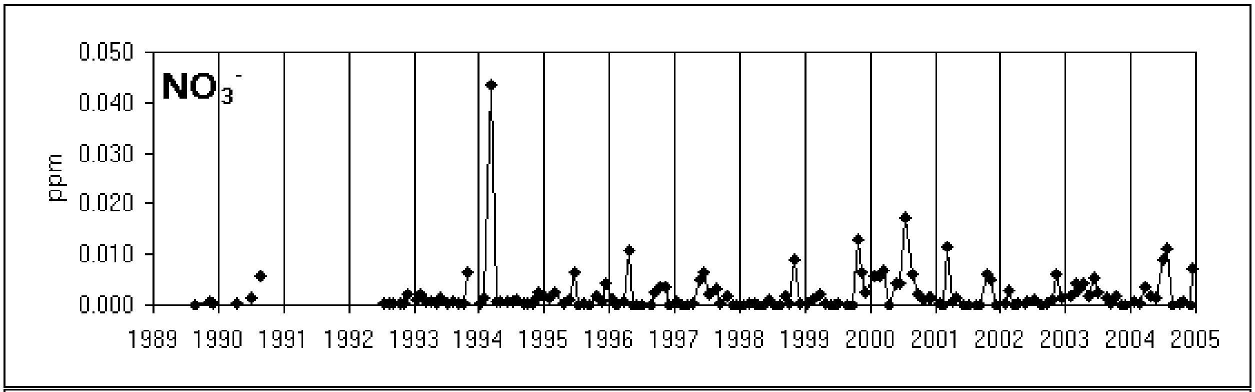

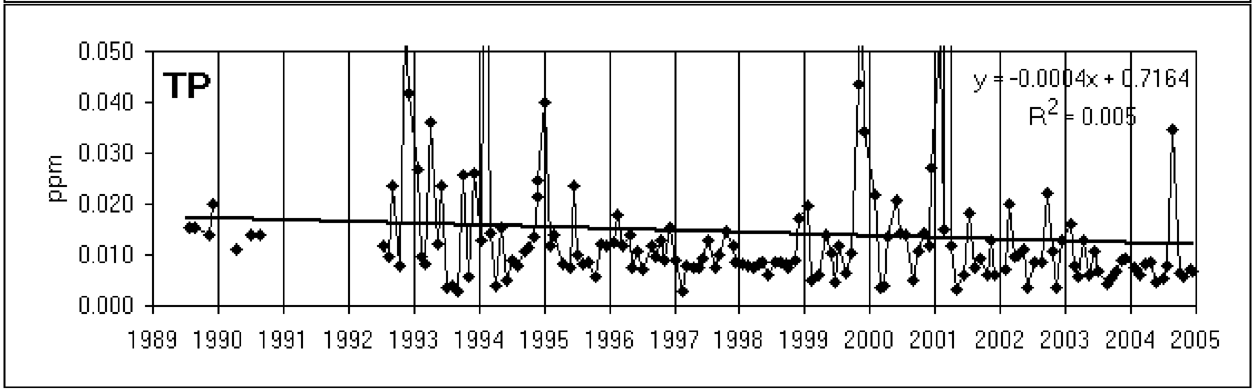

References

Southeast Environmental Research Center. 2025. Florida International University, Miami, FL USA: http://serc.fiu.edu/wqmnetwork/
